# Multidisciplinary Collaboration Mitigating Health Crises Rooted in Wildlife-Human Interaction

**DOI:** 10.3389/phrs.2024.1607266

**Published:** 2024-05-30

**Authors:** Afshin Abbaspour, Shahryar Sorooshian

**Affiliations:** ^1^ Prime School of Logistics, Saito University College, Petaling Jaya, Malaysia; ^2^ Department of Business Administration, University of Gothenburg, Gothenburg, Sweden

**Keywords:** multidisciplinary collaboration, health crises, zoonoses, wildlife-human interaction, one health

## Debate

In 1964, the term One Health was coined to emphasize the significance of integrating human and animal health in a unified framework. However, since then, there has been limited attention on implementing the One Health principles in real-world scenarios, assessing its effectiveness, and making necessary enhancements. Despite significant advances in science and technology, we remain vulnerable to infectious disease outbreaks, as evidenced by the recent COVID-19 pandemic. Many human diseases, including COVID-19, SARS, MERS, Ebola, and zoonotic influenza, among others, are actually zoonoses that originated or grew in the wild [1], and yet there is no (or very little) systematic or quantitative studies from management engineering perspective to address the issues of disease transmission from wildlife to humans. The COVID-19 pandemic, for example, has killed millions of people around the world. Global economic repercussions persist. When it comes to the effects of wildlife diseases on human health, the question is how to lessen the likelihood of future pandemics.

**FIGURE 1:**
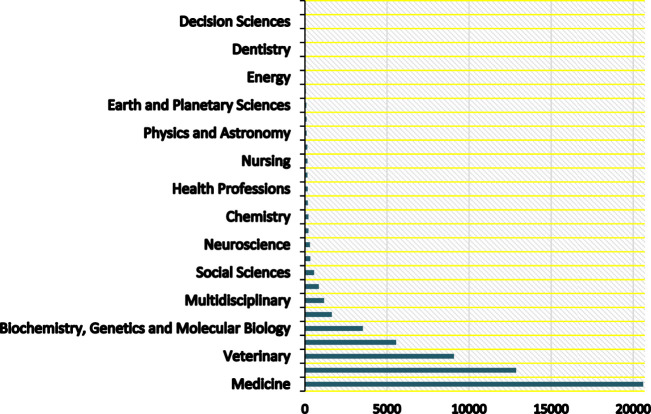
Research distribution by academic discipline (Multidisciplinary Collaboration Mitigating Health Crises Rooted in Wildlife-Human Interaction, Global trend, 2000–2024).

A path to improve public health has been mapped out by a number of scientific studies from a variety of fields. However, because each study has its own scope and perspective, implementing all of these methods on a global scale may result in a conflict of interest between different parts of society. To solve these intricate problems, we need to draw on the expertise of many fields and develop a unified system for global ecosystem monitoring and management. To identify zoonoses and stop them from spreading into widespread epidemics, we need a unified platform that integrates data and knowledge from many disciplines, including but not limited to medicine, biology, epidemiology, and the decision sciences. Academicians have already generated thousands of articles resulting from their comprehensive research, particularly in medicinal fields [2, 3]. However, limited attention has been given to bridging the gap between these academic pursuits and the managerial domain. Establishing a foundation for unified collaboration among various scientific disciplines is crucial, ensuring effective cooperation without the risk of interference in each other’s work. The authors initiated direct contact through email with a university professor from Scotland who is overseeing a constrained experiment on bat vaccination. The professor highlighted that a primary concern in Latin America hindering community adherence to bat vaccination is the concern about potential overpopulation of the species resulting from immunization. This underscores the fact that scientists conducting research in veterinary, medical or pharmaceutical fields should closely collaborate with management engineers or professionals from related fields. Such collaboration will assist authorities in determining, for instance, whether vaccinations help in controlling zoonotic diseases or overpopulated species like bats, which could potentially increase the likelihood of emerging novel zoonotic diseases. Such collaboration is essential to effectively communicate to the public and policymakers that their products are invaluable in mitigating the risk of future zoonotic diseases. The concept of “One Health” aims to develop unified and comprehensive methodologies for creating a healthier ecosystem for all living creatures. However, current efforts predominantly involve veterinary, biological, or medicinal approaches and solutions. This approach is comparable to manufacturers producing potentially useful products for individuals but lacking proper marketing strategies to introduce them to societies, resulting in the risk of bankruptcy or overstock. Figure 1, visualizes the archived articles of the Scopus database; the TITLE-ABS-KEY (zoonos*) search formula is used to find all documents whose titles, abstracts, or keywords contain any form of the word “zoonos”, capturing a wide range of literature on the subject of zoonotic diseases, from 2000 until April 2024. This is evidencing the unbalanced distribution of zoonoses research effort. The rabies vaccination program for bats [4] is just one example of a small-scale wildlife vaccination program that could be expanded and implemented in other parts of the world. Data collected on wildlife populations and microorganisms can be used for surveillance and early detection of emerging diseases [5]. This includes tracking different species or monitoring microorganisms that may cause infection. It takes a lot of time, effort, and money to find solutions, but in order to effectively implement them, governments and other stakeholders must be convinced of their necessity and allocate sufficient resources.

**FIGURE 2:**
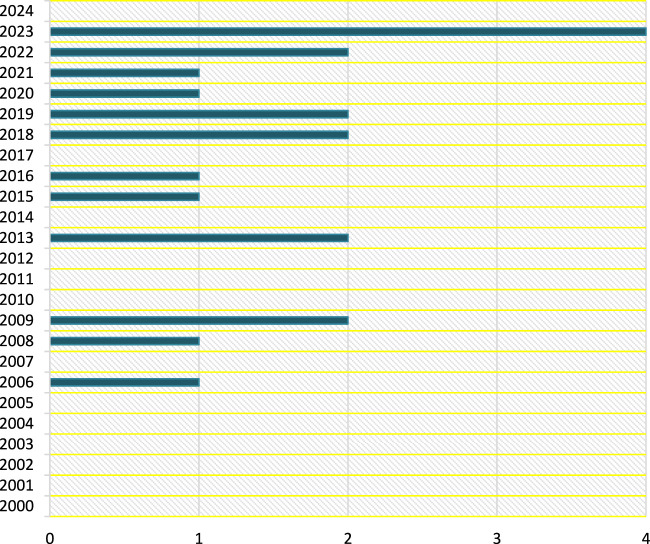
Decision Science contributions (Multidisciplinary Collaboration Mitigating Health Crises Rooted in Wildlife-Human Interaction, Global trend, 2000–2024).

Furthermore, this can only be accomplished by providing solid evidence and employing decision sciences to categorize the dangers posed by new diseases. Figure 2, however, visualizes the Scopus archived articles, from 2000 to 2024 and in decision sciences with TITLE-ABS-KEY (zoonos*) AND PUBYEAR > 1999 AND PUBYEAR < 2025 AND [LIMIT-TO (SUBJAREA, “DECI”)] search formula, incorporating keywords such as Office International des Epizooties, one health, zoonotic infectious disease surveillance, wildlife vaccination, and wildlife health surveillance.

A holistic understanding of many issues and effective solutions can be gleaned from combining data from various sources, such as social networks and government agencies. The non-intrusive nature of modern analysis tools like simulation software and the Metaverse platform has opened up new possibilities in healthcare systems. These resources can help scientists estimate and eliminate problems in real-world practices, as well as investigate and discover the best options for mitigating risks posed by wildlife diseases to human health [6]. However, manufacturers, the public, and government all need to be on board for these solutions to be fully implemented. Microbiologists may design vaccines to prevent disease in humans or animals, but manufacturers will only mass produce them if doing so is profitable [7]. In a similar vein, some people may refuse vaccination for personal reasons [8], and some governments may look into the effects of wildlife vaccination on ecosystems and human populations. Part human and part animal vaccinations may be the best solution to these problems, which necessitate the analysis of criteria related to objectives, the development of matrices to demonstrate the proportion of humans and wildlife creatures to be vaccinated in each part of the world and archived data to determine the relative importance of different stands and to justify decisions to all parties.

Currently, we find no specific examples to present here as a case to illustrate that scientists in the field of mathematics, management engineering, computer sciences, and etc., are actively engaged in the field of controlling infectious diseases. We have lack of knowledge on ethical consideration associated with wildlife consideration. There is lack of knowledge on how engage indigenous people in controlling such diseases and how make this cooperation feasible utilizing operations techniques. Decision sciences mainly act as a powerful tools in complex systems that predominantly use in industrial domains and we have lack of knowledge on how decision sciences can specifically contribute to addressing the challenges posed by wildlife diseases. The existing articles do not highlight the exact cause of obstacles to global cooperation and discuss strategies to overcome these barriers.

### Conclusion

**FIGURE 3:**
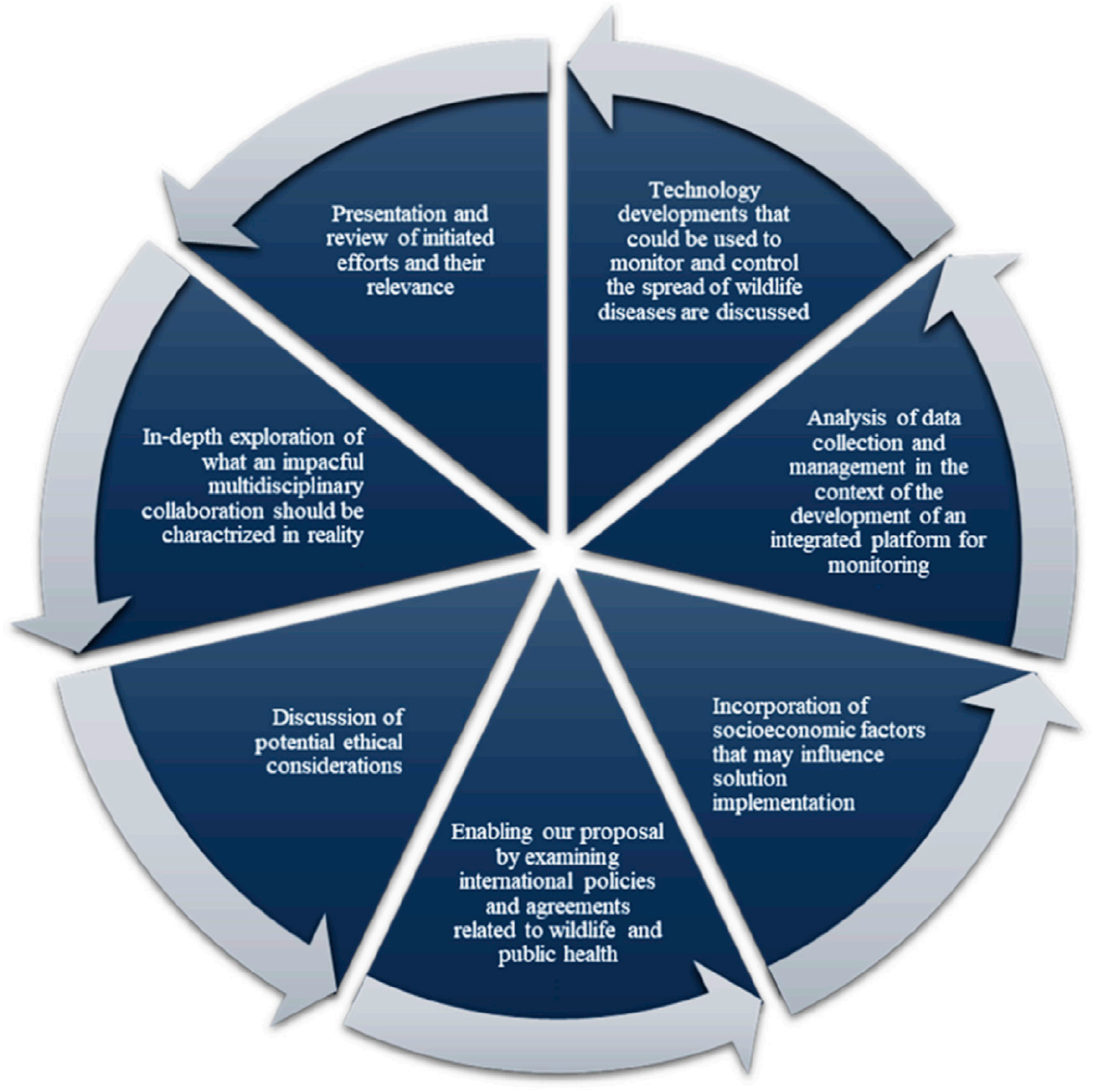
Visual recommendation derive from this study (Multidisciplinary Collaboration Mitigating Health Crises Rooted in Wildlife-Human Interaction, 2000–2024).

To sum up, a thorough and multidisciplinary strategy, illustrated in Figure 3, is required. This approach involves utilizing mathematical modelling, incorporating indigenous knowledge in our mathematical modelling and analyse the feasibility of the knowledge in controlling diseases, examining decision sciences in details, and analysing the efficiency of the proposed methodologies in the real world is necessary to prevent future pandemics and lessen the effects of wildlife diseases on human health. We require an integrated system unifying diverse database and expertise to detect and halt zoonotic diseases early, preventing global spread and epidemics. Collaboration and resource pooling can develop and enact strategies benefiting both human and animal populations. Employing decision science and technologies like extended realities can mitigate wildlife-related risks to human health.

If people, governments, and scientists all work together to implement these measures, we can lessen the devastating effects of wildlife diseases. Using data and objective criteria from multiple sources to overcome obstacles and defend decisions to all parties. To accomplish this, we can monitor wildlife health, populations, and microorganisms, and we can vaccinate wildlife on a local or global scale without serious adverse impacts on natural ecosystem [9, 10].
